# Characteristics of meta-analyses and their component studies in the *Cochrane Database of Systematic Reviews*: a cross-sectional, descriptive analysis

**DOI:** 10.1186/1471-2288-11-160

**Published:** 2011-11-24

**Authors:** Jonathan Davey, Rebecca M Turner, Mike J Clarke, Julian PT Higgins

**Affiliations:** 1 MRC Biostatistics Unit, Institute of Public Health, Cambridge, UK; 2 All-Ireland Hub for Trials Methodology Research, Centre for Public Health, Queen's University Belfast, Northern Ireland

## Abstract

**Background:**

Cochrane systematic reviews collate and summarise studies of the effects of healthcare interventions. The characteristics of these reviews and the meta-analyses and individual studies they contain provide insights into the nature of healthcare research and important context for the development of relevant statistical and other methods.

**Methods:**

We classified every meta-analysis with at least two studies in every review in the January 2008 issue of the *Cochrane Database of Systematic Reviews *(*CDSR*) according to the medical specialty, the types of interventions being compared and the type of outcome. We provide descriptive statistics for numbers of meta-analyses, numbers of component studies and sample sizes of component studies, broken down by these categories.

**Results:**

We included 2321 reviews containing 22,453 meta-analyses, which themselves consist of data from 112,600 individual studies (which may appear in more than one meta-analysis). Meta-analyses in the areas of gynaecology, pregnancy and childbirth (21%), mental health (13%) and respiratory diseases (13%) are well represented in the *CDSR*. Most meta-analyses address drugs, either with a control or placebo group (37%) or in a comparison with another drug (25%). The median number of meta-analyses per review is six (inter-quartile range 3 to 12). The median number of studies included in the meta-analyses with at least two studies is three (inter-quartile range 2 to 6). Sample sizes of individual studies range from 2 to 1,242,071, with a median of 91 participants.

**Discussion:**

It is clear that the numbers of studies eligible for meta-analyses are typically very small for all medical areas, outcomes and interventions covered by Cochrane reviews. This highlights the particular importance of suitable methods for the meta-analysis of small data sets. There was little variation in number of studies per meta-analysis across medical areas, across outcome data types or across types of interventions being compared.

## Background

Systematic reviews of randomized trials provide valuable evidence on the effectiveness of healthcare interventions, maximising power, minimising bias and avoiding undue emphasis on the results of individual studies. Many systematic reviews contain meta-analyses, in which the results from independent studies are formally combined using statistical methods. The *Cochrane Database of Systematic Reviews *(*CDSR*) is a major resource of systematic reviews on the effects of healthcare interventions that are periodically updated [1,2]. The reviews in the *CDSR *are prepared by members of The Cochrane Collaboration, which aims to make the findings from systematic reviews of interventions conveniently available so that healthcare consumers, professionals and providers can make choices about healthcare interventions using the most up-to-date and reliable evidence available on their relative effects [3]. The initiative is worldwide in its scope, involving input from more than 27,000 contributors in over 100 different countries, who work with the Cochrane Review Groups responsible for reviews in particular areas of health and health care [4]. As of 2011, more than 4,500 full Cochrane reviews have been produced and published protocols are available for nearly 2,000 more reviews that are at earlier stages in their development.

The objective of this paper is to report descriptive statistics relating to one complete issue of the *CDSR*, published in January 2008. We have classified each meta-analysis in the *CDSR *by types of interventions, type of outcome and medical specialty. In further work, the classifications will allow us to examine empirically the magnitude and variation of among-study heterogeneity in meta-analyses, enabling us to understand how certain characteristics influence the level of between-study heterogeneity. In this paper, we have described our methods for collating this collection of more than 22,000 meta-analyses and reported statistics relating to the interventions, outcomes and medical areas investigated in these meta-analyses. We have summarised the sizes of reviews, meta-analyses and studies in the *CDSR*, and explored how these vary across different settings.

A number of earlier papers have analysed large collections of systematic reviews and meta-analyses for other purposes. For example, Moher et al. [5] examined a cross-sectional sample of 300 systematic reviews (Cochrane and non-Cochrane) published in one month, and focused on their quality and reporting characteristics. A series of meta-epidemiological studies have analysed collections of meta-analyses in order to estimate the association between methodological flaws such as inadequate allocation concealment or inadequate blinding in randomised trials and exaggeration of the intervention effect; data from several of these studies were recently combined by Wood et al. [6]. Bow et al. [7] have described the characteristics of all Cochrane reviews relevant to child health, 1046 in total. To our knowledge, the characteristics of the entire Cochrane database have not previously been summarised, and the types of interventions and outcomes to which the meta-analyses within Cochrane reviews relate have not been examined in detail.

## Methods

**Figure 1:**
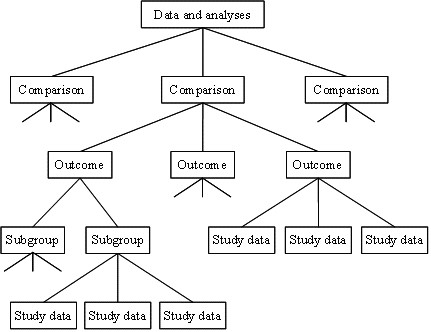
**Illustration of the hierarchy of a 'Data and analyses' section of a Cochrane review**. Reproduced with permission from Chapter 4 of the *Cochrane Handbook for Systematic Reviews of Interventions*.

The full content of the first issue from 2008 of the *CDSR *was provided to us by the Nordic Cochrane Centre. The highly structured nature of every Cochrane review allowed us to focus on the sections of each review covering 'Data and analyses', and on any pair-wise comparisons of interventions reported and the outcomes within these. Each outcome could contain several subgroups of studies, and each subgroup could contain data from several studies. This structure is illustrated in Figure 1.

### Eligibility criteria

We were interested in all full reviews of the effects of health or social care interventions published in Issue 1, 2008 of the *CDSR *that reported at least one eligible meta-analysis (see below). We excluded protocols for reviews, reviews from the Cochrane Methodology Review Group, and reviews that had been marked as withdrawn.

We defined an eligible meta-analysis as a forest plot that had been generated using the Review Manager (RevMan) software [8], and that included data from at least two different studies. We discarded forest plots with a single study, since a meta-analysis would not be performed on a single study, and because Cochrane review authors are often discouraged from including forest plots when there is only one study, meaning that any such forest plots published in Cochrane reviews would not be a representative sample. We included forest plots irrespective of whether review authors had elected to display the results of the meta-analysis within them. In some forest plots, the subgroups presented within an outcome were not mutually exclusive, since the same study data may be included in more than one subgroup. We therefore checked as far as possible for study duplications and extracted data for only the first occurrence of each study in each forest plot.

The *CDSR *includes four types of data which systematic review authors have extracted from the included studies: binary data, continuous data, generic results and "O-E and variance" data. Binary data and continuous data have been entered directly into RevMan as numbers of events or means and standard deviations, together with numbers of participants in each intervention group in each study. Generic results have been entered as an effect estimate with corresponding standard error for each study, and "O-E and variance" data have been entered as the observed-minus-expected number of events and variance for each study. Generic results include time-to-event outcomes (e.g. analysed as hazard ratios) and ordinal outcomes (e.g. analysed as odds ratios), as well as binary or continuous data from studies with complex designs, while "O-E and variance" data are typically derived from time-to-event outcomes. We analysed these latter two data types together as mixed outcome data.

**Figure 2:**
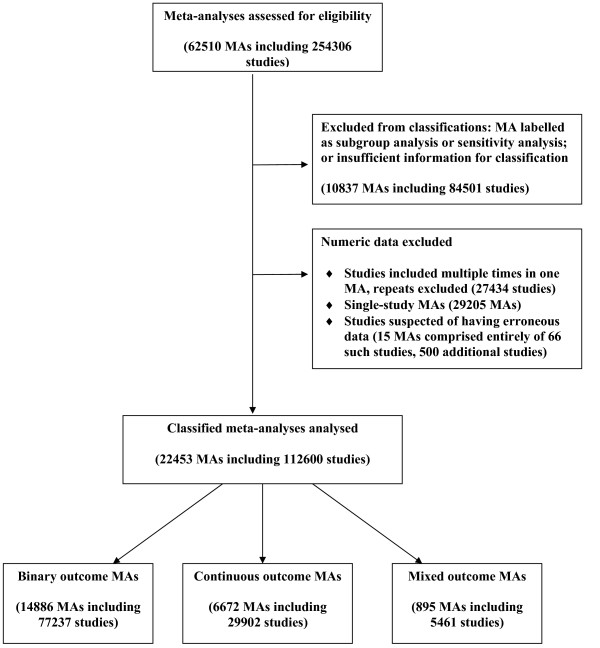
**Flow diagram showing eligibility and exclusions of meta-analyses and studies**.

There were occasional mistakes in how the review data had been entered into the hierarchy of the database, one common example being subgroups listed as if they were separate outcomes. A handful of reviews did not provide the names of the outcome measures to which their reported meta-analyses related. Incorrectly entered review information (be it at the level of individual meta-analyses or whole reviews) was corrected manually wherever possible, or excluded if the appropriate form of the data was impossible to determine. Furthermore, we excluded data from the large number of studies in the database that appeared to have a sample size of 2, with 0/1 events recorded in each group for studies with binary outcomes, means and standard deviations of 0 in each group for studies with continuous outcomes, or O-E and variance of 0 for studies with "O-E and variance" data. These data patterns arose because of the way that forest plots in the version of RevMan used up to 2008 stored information on studies that were listed in the plot but without contributing data to the specific meta-analysis, and it is likely that almost all of these studies do not represent genuine data. Studies of sample size 2 with a different data pattern recorded were not excluded. The flow diagram in Figure 2 illustrates where and why meta-analyses or studies were excluded.

### Classification schemes

Each eligible meta-analysis within each review was classified by medical specialty, types of interventions involved in the pair-wise comparison and type of outcome. To classify medical specialties, we used 11 categories from a taxonomy [9] developed by the UK National Institute for Health and Clinical Excellence (NICE) (see Table 1). Our classification scheme for interventions was based on the Health Research Classification System [10] developed by the UK Clinical Research Collaboration (UKCRC). Our outcome categories were based on those used by Wood [11], and the Foundation for Health Services Research [12]. During a pilot study (see below), some amendments to our initial schemes were made. Our final classifications contained 17 categories for type of intervention (15 'active' and two 'control') and 23 categories for type of outcome (Tables 2 and 3). Two general outcome categories were included: "infection/onset of new acute/chronic disease" and "symptoms/signs reflecting continuation or end of condition". These categories cover the many outcomes with loose descriptions such as "absence of disease" or "no improvement" where the method of outcome measurement is unclear.

**Table 1:** Distribution of medical specialties for the 22453 eligible meta-analyses in our sample

Medical Specialty	Frequency	
Cancer	905	(4%)
Cardiovascular	1693	(8%)
Central nervous system/musculoskeletal	1965	(9%)
Digestive/endocrine, nutritional and metabolic	2704	(12%)
Gynaecology, pregnancy and birth	4656	(21%)
Infectious diseases	965	(4%)
Mental health and behavioural conditions	2918	(13%)
Pathological conditions, symptoms and signs	701	(3%)
Respiratory diseases	3021	(13%)
Urogenital	1289	(6%)
Other^1^	1637	(7%)

Total	22453	

^1 ^Other: Blood and immune system, Ear and nose, Eye, General health, Genetic disorders, Injuries, accidents and wounds, Mouth and dental, Skin

**Table 2:** Distribution of Pair-wise Interventions in meta-analyses in the *CDSR*

	Second Intervention					
	
First Intervention	vs. Control	vs. Placebo	vs. Pharm	Vs. Non-Pharm^1 ^from same category	vs. Non-Pharm^1 ^from different category	Total
Pharmacological	4027 (18%)	4321 (19%)	5571 (25%)	n/a	186 (<1%)	14105 (63%)
Vaccines	95 (<1%)	113 (<1%)	0 (0%)	49 (<1%)	0 (0%)	257 (1%)
Surgical	241 (1%)	2 (<1%)	29 (<1%)	1204 (5%)	86 (<1%)	1562 (7%)
Medical devices	706 (3%)	50 (<1%)	82 (<1%)	941 (4%)	159 (<1%)	1938 (9%)
Cellular and gene	29 (<1%)	6 (<1%)	0 (0%)	57 (<1%)	7 (<1%)	99 (<1%)
Radiotherapy	124 (<1%)	65 (<1%)	3 (<1%)	56 (<1%)	63 (<1%)	311 (1%)
Physical	318 (1%)	10 (<1%)	8 (<1%)	72 (<1%)	39 (<1%)	447 (2%)
Educational	303 (1%)	4 (<1%)	1 (<1%)	34 (<1%)	27 (<1%)	369 (2%)
Behavioural	380 (2%)	7 (<1%)	3 (<1%)	165 (<1%)	14 (<1%)	569 (3%)
Screening	17 (<1%)	0 (0%)	0 (0%)	0 (0%)	0 (0%)	17 (<1%)
Complementary	216 (1%)	104 (<1%)	41 (<1%)	8 (<1%)	19 (<1%)	388 (2%)
Psychological	379 (2%)	1 (<1%)	6 (<1%)	116 (<1%)	44 (<1%)	546 (2%)
Resources and infrastructure	506 (2%)	0 (0%)	14 (<1%)	52 (<1%)	33 (<1%)	605 (3%)
Complex	505 (2%)	36 (<1%)	43 (<1%)	200 (<1%)	154 (<1%)	938 (4%)
Other	188 (1%)	44 (<1%)	8 (<1%)	51 (<1%)	11 (<1%)	302 (1%)

Total	8034 (36%)	4763 (21%)	5809 (26%)	3005 (13%)	842 (4%)	22453

^1^Non-Pharm = all intervention categories as listed for the First intervention, other than Pharmacological

n/a = not applicable

**Table 3:** Distribution of Outcomes in meta-analyses in the *CDSR*

Outcome	Frequency	
Signs/symptoms reflecting continuation/end of condition	3639	(16%)
Adverse events	2368	(11%)
Infection/onset of new acute/chronic disease	2215	(10%)
Biological markers (e.g. VO_2 _max)	2071	(9%)
General physical health (e.g. heart rate)	1921	(9%)
Obstetric outcomes	1477	(7%)
All-cause mortality	1278	(6%)
Hospital stay/process measures	1158	(5%)
Quality of life/functioning	958	(4%)
Pain	802	(4%)
Mental health outcomes	743	(3%)
Surgical/device related success/failure	737	(3%)
Withdrawals/dropouts	643	(3%)
Internal structure (e.g. radiograph outcomes)	504	(2%)
Major morbidity event (e.g. stroke)	502	(2%)
Composite (at least 1 non-mortality/morbidity)	460	(2%)
Composite (mortality/morbidity only)	224	(1%)
Cause-specific mortality	194	(1%)
Consumption (e.g. use of beta2 agonists)	190	(1%)
External structure (e.g. eczema)	160	(1%)
Other	94	(0.4%)
Satisfaction with care	86	(0.4%)
Resource use	29	(0.1%)

Total	22453	

### Pilot study

A pilot study was built into our research. We sampled one Cochrane review from each of the 49 Cochrane Review Groups (excluding the Cochrane Methodology Review Group) from Issue 4, 2007 of the *CDSR*. The meta-analyses in these reviews were independently classified for outcomes and interventions by three people (JD, RMT and JPTH). Disagreements were discussed and used to refine the lists of categories for the main study.

### Data collection and management

The *CDSR *data for Issue 1, 2008 were imported into Microsoft^® ^Office Access. To facilitate classifications, relevant summary information for each systematic review were extracted into a Microsoft Access form, drawing on various hard-coded elements of a Cochrane review [Box 2.2.b in Cochrane Handbook [3]]. In particular, the form provided rapid access to (i) the review's title; (ii) short text descriptions of interventions and outcomes from the captions of each forest plot; (iii) detailed review eligibility criteria for participants, interventions and outcomes; (iv) objectives, selection criteria and results from the review's abstract; and (v) descriptions of participants, interventions and outcomes for up to five studies with the highest statistical weight in the first meta-analysis of the review. Reviews with no meta-analyses, and forest plots with fewer than two studies were not extracted into the Access forms.

Classifications were performed directly into the Access form by a single person (JD), who consulted other authors (JPTH, RMT) when uncertain of the most appropriate choice. In practice, classifications of interventions and outcomes were based primarily on review titles and short text captions from forest plots. The outcome categories were ordered so that outcomes potentially falling under multiple categories would be classified as the category highest on the list. Classification of medical specialties was performed by a single person (MJC), and was usually based on the review title, with the abstract consulted if necessary.

### Statistical analysis

We present tabular descriptive statistics for various characteristics of the eligible meta-analyses in *CDSR*. We summarise how the database is comprised with regards to pair-wise intervention categories, outcome categories and medical specialty areas. We present descriptive results pertaining to the reviews (number of meta-analyses and number of comparisons per review), comparisons (number of outcomes per comparison), meta-analyses (number of studies per meta-analysis) and studies (sample size of individual studies). We also describe the relationships between these characteristics and the different category groupings used for interventions, outcomes and medical specialties.

## Results

Of a total of 5171 records in the *CDSR *(Issue 1, 2008), 3385 were reviews rather than protocols, and 2492 of these met our eligibility criteria and contained at least one eligible meta-analysis of two or more studies. Following the removal of ineligible meta-analyses (Figure 2), we were left with 2321 reviews, which contained 22,453 meta-analyses. These meta-analyses incorporate data from 112,600 studies.

### Types of medical specialty

The medical specialties of the 22,453 meta-analyses are given in Table 1. The category "Gynaecology, pregnancy and birth" is the most frequently occurring, accounting for over a fifth (21%) of all meta-analyses. This reflects the fact that the Pregnancy and Childbirth Group and the Neonatal Group are two of the four Cochrane Review Groups which have produced the highest numbers of reviews. The two next largest categories are "Respiratory diseases" (13%) and "Mental health and behavioural conditions" (13%). The variation across medical areas partly reflects differences in longevity among the Cochrane Review Groups, as well as differences in research activity.

### Types of interventions

Each meta-analysis makes a comparison of two interventions, and each of the interventions was categorised separately, with the initial intervention in the pair being considered the "active" intervention and the second intervention being the "comparator". The distribution of the comparisons made in the 22,453 meta-analyses is summarised in Table 2. As one might expect in a database of reviews of the effects of health care, the *CDSR *is dominated by pharmacological interventions. The initial intervention listed is pharmacological in just under two-thirds (63%) of all pair-wise interventions examined. In addition to its primary importance as an active intervention, the pharmacological category also comprises over a quarter (26%) of all comparator interventions in the *CDSR*. The most common pair-wise comparison is of a pharmacological versus a pharmacological intervention (25%), followed by pharmacological versus placebo (19%) and pharmacological versus control (18%). These three pair-wise interventions account for over 60% of all comparisons in the *CDSR*. The distinction between placebo and control might not be an accurate representation, since review authors may have coded placebo as 'control' when specifying their interventions. Combining the placebo and control comparators suggests that 37% of meta-analyses evaluated the fundamental efficacy of a pharmacological intervention.

Overall, the most common comparator intervention is control (which we defined as a control intervention which is not explicitly labelled or described as placebo; examples include "usual care" and "no treatment"). Control groups are present in over one third (36%) of all pair-wise comparisons. Placebos account for over one fifth (21%) of comparators and are predominantly paired with active pharmacological interventions: 4321/4763 (91%) of all placebo-controlled comparisons relate to pharmacological rather than non-pharmacological interventions.

Comparisons of a non-pharmacological intervention with an intervention from the same category are reasonably common in the *CDSR *(13%). These are principally comparisons between surgical procedures (5%) or medical devices (4%). Surgical procedures in particular are frequently compared with another from the same intervention category; 1204 of the 1562 surgical meta-analyses (77%) compared two surgical procedures. Medical devices (9%) and surgery (7%) are the two most frequently occurring initial intervention types after pharmacological interventions.

### Types of outcome

Table 3 presents the types of outcomes assessed in the 22,453 meta-analyses. The largest outcome category is signs or symptoms reflecting the continuation or end of a medical condition (16%). This is a broad category, which includes commonly recorded outcomes such as "presence/absence of disease" (in a non-preventative review) and "clinical improvement". These types of outcome are routinely measured in a large number of healthcare areas. Following this in order of size is the category of adverse events (11%). Many outcomes included in this category were simply labelled "adverse events", while some had a more specific label such as "adverse events: weight gain", and other outcomes such as "headache" were assigned to this category if the review title and objectives suggested they related to side effects rather than the main effect of the intervention. Another large category is infection or onset of a new acute or chronic disease (10%), which is largely comprised of binary outcomes from reviews which investigated whether treatments aimed at prevention of a particular illness had succeeded or failed.

Biological markers (9%) include quantifiable biological parameters, typically measured in a laboratory, such as blood components (e.g. CD4 count). General physical health measures (9%) include all physical health measurements manually assessed by the clinician. This includes routine measures such as heart rate or BMI. Obstetric outcomes (7%) are heavily represented by the two afore-mentioned, large Cochrane Review Groups covering pregnancy, childbirth and neonatal care. This category includes binary events such as becoming pregnant or having a miscarriage, as well as continuous outcomes such as fetal measurements or information on gestational age. The most objectively defined category is all-cause mortality, which comprises 6% of the outcomes. These outcomes were usually labelled as "All-cause mortality", but we also assigned phrases such as "Survival" and "Neo-natal mortality" to this category.

The seven categories discussed above represent two-thirds of all the outcomes in the *CDSR*, with the remaining third split between the other 16 outcome categories (Table 3).

### Composition of systematic reviews

The majority (61%) of the 2321 reviews that contained at least one eligible meta-analysis included only one pair-wise comparison of interventions (Table 4). 86% of reviews measured outcomes for three or fewer comparisons and only 4% looked at seven or more. On closer examination of a review which appeared to report meta-analyses for the largest number of comparisons, some of the 23 comparisons were found to relate to different methods of combining data rather than separate pair-wise comparisons of interventions. The largest number of genuinely different pair-wise comparisons was 20, in a review comparing the effectiveness of interventions for preventing hypotension in women having Caesarean section under spinal anaesthesia [13].

**Table 4:** Number of comparisons per review, outcomes per comparison and meta-analyses per review in the *CDSR*.

	Min	25%	Median	75%	Max
Number of comparisons in 2321 reviews	1	1	1	2	23
Number of outcomes in 4755 comparisons	1	1	3	6	68
Number of meta-analyses in 2321 reviews	1	3	6	12	128

The median number of outcomes per comparison was three (inter-quartile range 1 to 6; Table 4). In 25% of all 4755 comparisons, only one outcome was reported. Several reviews included large numbers of outcomes relating to the same comparison, with 17% of all reviews including at least one comparison that looked at more than ten outcomes.

Of the 2321 reviews in the data set (which all included at least one meta-analysis of two or more studies), just under 10% contained only one meta-analysis. At the other end of the spectrum, one in ten reviews contained 22 or more meta-analyses. We note again that forest plots reporting results for two or more studies were regarded as meta-analyses, irrespective of whether review authors had elected to display the meta-analysis results. The median number of meta-analyses included in a review was six (inter-quartile range 3 to 12). The distribution exhibits positive skew, with five reviews examining more than 100 meta-analyses, the maximum being 128, in a review comparing immunosuppressive regimens for treating kidney transplant recipients [14].

### Number of studies per meta-analysis

In our sample of 22,453 meta-analyses from the *CDSR*, which needed to contain at least two studies to be eligible, the median number of included studies was three (inter-quartile range 2 to 6; see Table 5). Over a third (36%) of the meta-analyses included the minimum requirement of two studies only, and just under three quarters (75%) contained five or fewer studies.

**Table 5:** Number of studies per meta-analysis, overall and broken down by outcome type, intervention comparison type and medical area

		Meta-analyses	**50%**^1^	75%	90%	99%	Max
*All*	Total	22453	3	6	10	28	294

*Data type*	Dichotomous	14886	3	6	10	28	294
	Continuous	6672	3	5	8	24	98
	Mixed	895	4	7	12	46	133

*Medical specialty*	Cancer	905	5	8	14	43	69
	Cardiovascular	1693	4	7	13	38	58
	Central nervous system/musculoskeletal	1965	3	5	8	21	49
	Digestive/endocr., nutritional and metabolic	2704	4	6	11	31	138
	Gynaecology, pregnancy and birth	4656	3	5	8	20	76
	Infectious diseases	965	3	5	9	27	63
	Mental health and behavioural conditions	2918	3	5	10	30	174
	Pathological conditions, symptoms and signs	701	3	6	10	31	294
	Respiratory diseases	3021	3	5	8	20	39
	Urogenital	1289	3	5	9	21	31
	Other	1636	3	6	10	34	133

*Interventions*	Pharm. vs. Control/Placebo	8348	3	6	10	30	294
	Pharm. vs. Pharm.	5571	3	6	10	28	174
	Pharm. vs. Non-Pharm.	424	3	5	11	25	36
	Non-Pharm. vs. Control/Placebo	4449	3	5	9	25	61
	Non-Pharm. vs. Non-Pharm.	3661	3	5	9	28	58

*Outcomes*	Signs/symptoms reflecting continuation/end of condition	3639	3	5	9	29	110
	Adverse events	2368	3	6	10	26	270
	Infection/onset of new acute/chronic disease	2215	3	6	10	24	294
	Biological markers	2071	3	6	10	28	138
	General physical health	1921	3	5	9	27	58
	Obstetric outcomes	1477	3	6	11	24	76
	All-cause mortality	1278	4	8	13	36	75
	Resource use/hospital stay/process	1187	3	5	9	27	99
	Cause-specific mortality/major morbidity event/composite (mortality or morbidity)	920	4	6	11	34	133
	Other outcomes (semi-objective)^2^	2044	3	6	11	30	174
	Other outcomes (subjective)^3^	3239	3	5	9	23	69

^1^Minimum values and 25^th ^percentiles are not listed, since these were identical across most meta-analysis types. The minimum value was 2 in all rows (noting that meta-analyses needed to contain at least 2 studies to be eligible for inclusion). The 25^th ^percentile was 3 for Cancer and All-cause mortality, and 2 in every other row.

^2^Other outcomes (semi-objective): External structure, Internal structure, Surgical/device related success/failure, Withdrawals/drop-outs

^3^Other outcomes (subjective): Pain, Mental health outcomes, Quality of life/functioning, Consumption, Satisfaction with care, Composite (at least 1 non-mortality/morbidity)

Some of the more widely studied medical specialty areas in the *CDSR *include meta-analyses that are able to draw upon a wealth of studies, the largest containing 294 [15], whilst 1% of meta-analyses contain 28 studies or more. Among the 11 specialty categories we used, cancer had a slightly higher median number of included studies (5) than any of the other categories.

There is no clear evidence to suggest that the number of studies per meta-analysis is strongly related to the outcome data type, or to the types of interventions being compared. Meta-analyses of all-cause mortality appear to contain slightly larger numbers of studies than other types of outcome. A Wilcoxon rank-sum test comparing the numbers of studies in meta-analyses of all-cause mortality vs. all other outcome types gave a P-value of 0.001; however, this analysis was not pre-specified and should be interpreted with caution.

### Study sample size

Sample size of individual studies varies considerably across reviews and meta-analyses in the *CDSR *(Table 6): from very small studies containing only two individuals, up to some very large studies aiming to investigate the efficacy of vaccines or the impact of screening, which contained hundreds of thousands or even millions of individuals. The overall mean sample size for studies in the *CDSR *is 513. However, the distribution of sample sizes is better summarised by the median of 91 and the inter-quartile range of 44 to 210.

**Table 6:** Study sample size, overall and broken down by outcome type, intervention comparison type and medical area

		Studies	25%	50%	75%	Max
*All*	Total	112600	44	91	210	1242071

*Data type*	Dichotomous	77237	50	102	243	1242071
	Continuous	29902	33	62	142	18851
	Mixed	5461	21	86	251	36511

*Medical specialty*	Cancer	6441	72	154	326	266064
	Cardiovascular	10686	44	102	289	67800
	Central nervous system/musculoskeletal	8299	43	80	149	9440
	Digestive/endocr., nutritional and metabolic	15345	42	79	160	18819
	Gynaecology, pregnancy and birth	20098	60	118	293	23697
	Infectious diseases	4502	59	130	371	419748
	Mental health and behavioural conditions	15218	36	63	165	9020
	Pathological conditions, symptoms and signs	4448	40	61	115	2463
	Respiratory diseases	13153	36	87	227	82892
	Urogenital	5914	39	71	153	4912
	Other	8496	44	90	198	1242071

*Interventions*	Pharm. vs. Control/Placebo	44156	41	90	222	58050
	Pharm. vs. Pharm.	28018	48	99	222	25180
	Pharm. vs. Non-Pharm.	2018	50	74	135	7711
	Non-Pharm. vs. Control/Placebo	20892	41	92	229	1242071
	Non-Pharm. vs. Non-Pharm.	17516	45	85	169	82892

*Outcomes*	Signs/symptoms reflecting continuation/end of condition	17621	42	83	197	359600
	Adverse events	11898	49	100	242	82892
	Infection/onset of new acute/chronic disease	11284	56	110	258	1242071
	Biological markers	10578	35	64	147	266064
	General physical health	8959	30	66	177	131271
	Obstetric outcomes	7376	60	117	293	18453
	All-cause mortality	8302	52	110	281	259627
	Resource use/hospital stay/process	5454	50	98	200	419748
	Cause-specific mortality/major morbidity event/composite (mortality or morbidity)	5413	69	165	449	266064
	Other outcomes (semi-objective)^1^	10886	44	81	177	131271
	Other outcomes (subjective)^2^	14490	38	71	162	359600

^1^Other outcomes (semi-objective): External structure, Internal structure, Surgical/device related success/failure, Withdrawals/drop-outs

^2^Other outcomes (subjective): Pain, Mental health outcomes, Quality of life/functioning, Consumption, Satisfaction with care, Composite (at least 1 non-mortality/morbidity)

Studies reporting dichotomous data have a median size of 102 and an inter-quartile range of 50 to 243, whereas studies reporting continuous data have a lower median size (62) and inter-quartile range (33 to 142). This may be because continuous outcomes (e.g. blood pressure or change in peak expiratory flow) tend to be more complex and labour-intensive to measure, making them unsuitable as outcomes in very large studies. On the other hand, dichotomous outcomes such as presence or absence of disease can be collected in a quick and more efficient manner for large numbers of individuals. In addition, statistical power tends to be higher for continuous outcomes than for dichotomous outcomes, so sample size calculations will generally lead to lower sample sizes when the primary outcome is continuous.

Study sizes show notable variation across medical specialties. The medians and quartiles are highest in cancer, and high also for meta-analyses in the areas of infectious diseases and gynaecology, pregnancy and birth. Study sizes tend to be lower in the areas of mental health and behavioural conditions and pathological conditions, symptoms and signs.

There is relatively little variation in medians and inter-quartile ranges of sample size across different types of intervention comparison. However, the maximum sample size for pair-wise interventions comparing non-pharmacological interventions against control or placebo is substantially larger than for the other categories. This can be explained by the fact that the largest studies such as those involving vaccines or screening are included under this category.

Across outcome types, sample sizes are highest for the category including cause-specific mortality, major morbidity events and composite mortality/morbidity events, and are lowest for biological markers and general physical health measures.

## Discussion

The *CDSR *provides high quality evidence on the effects of healthcare interventions and is widely cited in all areas of medicine. To our knowledge, we report the largest study which has classified and described the characteristics of the entire collection of meta-analyses contained within the *CDSR*, although many previous studies have explored characteristics of subsets of the *CDSR*. Our descriptive analysis provides information on the frequencies of different outcome types, intervention types and medical specialties in the *CDSR*, and summarizes the distributions of meta-analysis and study size, overall and within different settings.

Our results show that the majority of meta-analyses in the *CDSR *evaluate pharmacological interventions, either in comparison with placebo or control (37%) or in comparison with another pharmacological intervention (25%). The most frequently examined non-pharmacological interventions were medical devices (9%) and surgical procedures (7%). The number of studies per meta-analysis was typically very small, with an overall median of 3 (inter-quartile range 2 to 6). When a meta-analysis includes very few studies, it is difficult to estimate the between-study variance, and researchers may choose to summarize the results narratively only. Meta-analyses containing 5 or fewer studies are generally viewed as small meta-analyses, in which statistical synthesis may be problematic [16,17], and almost 75% of meta-analyses in the *CDSR *fall into this category. However, our analyses include all data presented in forest plots, even where the review authors had chosen not to display the result of the meta-analysis, and numbers of studies are likely to be lower in these cases than in meta-analyses where the result was displayed.

The distribution of number of studies per meta-analysis was consistent across many of the different outcome types, intervention comparison types and medical specialties. Meta-analyses in cancer tended to include slightly more studies compared with meta-analyses in other medical areas, as did meta-analyses of all-cause mortality compared with those investigating other outcomes. There was much greater variation across the categories in the distribution of the sample sizes of individual studies. Sample sizes were lower for studies measuring labour-intensive outcome types such as biological outcomes and tended to be higher for studies of dichotomous outcomes such as mortality and morbidity events. Sample sizes also varied substantially across medical specialties.

One limitation of our study is that classifications of meta-analysis characteristics were carried out by only one assessor. Due to the very large scale of the study, the classification work required several months for completion and it was not possible for two authors to undertake this. However, cases in which the appropriate categorisation was unclear were discussed with one of the other authors, and steps were taken to ensure consistency across the classifications. Extraction of the data was automated, since manual extraction was not practical for such a large data set, so our analyses relied on the information that had already been entered by the review authors. Efforts were made to exclude duplications of the same study within a meta-analysis, where the meta-analysis included subgroups which were not mutually exclusive. Identification of duplicates was based on the study identifier appearing twice in an identical form, and it is possible that some duplicates were missed through being labelled slightly differently (for example as "Smith 2001a" and "Smith 2001b"). Variation in the labelling of studies across Cochrane reviews meant that manual data extraction would be required to obtain a unique identifier for each study; therefore we were unable to examine overlap among different meta-analyses. Within reviews, studies often contribute data to multiple meta-analyses relating to different outcomes and comparisons. Studies may also appear in multiple reviews, either contributing identical data or contributing data for different outcomes and comparisons. The descriptive analyses of study sample size (Table 6) should therefore be interpreted with caution, since some studies are over-represented in the data set.

This research offers an insight into the characteristics of the meta-analyses which make up the *CDSR*. In other areas of this research project we have used these data to investigate the distribution of between-study heterogeneity across meta-analyses, and explore which meta-analysis characteristics influence the degree of heterogeneity [18]. In later work, we plan to explore the factors which influence the degree of inconsistency among studies [19].

## Conclusions

It is clear that the numbers of studies eligible for meta-analyses are typically very small for all medical areas, outcomes and interventions covered by Cochrane reviews. This highlights the particular importance of suitable methods for the meta-analysis of small data sets. There was little variation in number of studies per meta-analysis across medical areas, across outcome data types or across types of interventions being compared. Sample sizes of individual studies within meta-analyses varied considerably across reviews and meta-analyses. Among medical specialties, study sizes were found to be highest in meta-analyses related to cancer, infectious diseases, or gynaecology, pregnancy and birth, and lowest in the areas of mental health and behavioural conditions and pathological conditions, symptoms and signs.

## Competing interests

The authors declare that they have no competing interests.

## Authors' contributions

JD developed and maintained the Access database; undertook the classification of outcomes and interventions; performed the analyses; contributed to the interpretation; and drafted the manuscript. RMT prepared the protocol for the project; participated in classifications, analyses and interpretation; and critically edited the manuscript. MJC contributed to development of the protocol; undertook the classification of clinical areas; contributed to the interpretation; and critically edited the manuscript. JPTH conceived the study; contributed to development of the protocol; participated in classifications; contributed to the interpretation; and critically edited the manuscript. All authors read and approved the final manuscript.

## Pre-publication history

The pre-publication history for this paper can be accessed here:

http://www.biomedcentral.com/1471-2288/11/160/prepub
